# Effect of Graphene-Graphene Oxide Modified Anode on the Performance of Microbial Fuel Cell

**DOI:** 10.3390/nano6090174

**Published:** 2016-09-15

**Authors:** Na Yang, Yueping Ren, Xiufen Li, Xinhua Wang

**Affiliations:** Jiangsu Key Laboratory of Anaerobic Biotechnology, Jiangsu Cooperative Innovation Center of Technology and Material of Water Treatment, School of Environmental and Civil Engineering, Jiangnan University, Wuxi 214122, Jiangsu, China; yangna191991@126.com (N.Y.); xhwang@jiangnan.edu.cn (X.W.)

**Keywords:** graphene, graphene oxide (GO), microbial fuel cell (MFC), modified anode, hydrophilicity

## Abstract

The inferior hydrophilicity of graphene is an adverse factor to the performance of the graphene modified anodes (G anodes) in microbial fuel cells (MFCs). In this paper, different amounts of hydrophilic graphene oxide (GO) were doped into the modification layers to elevate the hydrophilicity of the G anodes so as to further improve their performance. Increasing the GO doped ratio from 0.15 mg·mg^−1^ to 0.2 mg·mg^−1^ and 0.25 mg·mg^−1^, the static water contact angle (*θ*_c_) of the G-GO anodes decreased from 74.2 ± 0.52° to 64.6 ± 2.75° and 41.7 ± 3.69°, respectively. The G-GO_0.2_ anode with GO doped ratio of 0.2 mg·mg^−1^ exhibited the optimal performance and the maximum power density (*P*_max_) of the corresponding MFC was 1100.18 mW·m^−2^, 1.51 times higher than that of the MFC with the G anode.

## 1. Introduction

Microbial fuel cell (MFC) is an emerging technology that converts chemical energy of organic pollutants directly into electricity with electro-active microorganisms as catalysts [1,2]. However, the low power density of MFCs has long been one of the critical problems that limited their practical applications in microbial sensors, biological remediation and wastewater treatment [3,4]. In MFCs, anodes are the habitats of the microorganisms and their hydrophilicity is a significant factor that influences the attachment of the microorganisms and the formation of highly active biofilms [5,6]. Guo and coworkers [7] reported that glassy carbon (GC) anodes modified by hydrophilic functional groups (-N^+^(CH_3_)_3_, -SO_3_^−^ or -OH) exhibited faster initial bacterial adhesion and higher anode biomass than the hydrophobic GC anode (modified by -CH_3_). Moreover, the current densities of the hydrophilic -N^+^(CH_3_)_3_, -SO_3_^−^ or -OH functionalized GC anodes are more than 2.5 times higher than that of the -CH_3_ functionalized GC anode. In the last couple of years, the positive effect of the graphene modified anodes (G anodes) on promoting the electricity generation performance of MFCs has been verified [8,9]. However, the inferior hydrophilicity of graphene is an adverse factor to the performance of the G anodes. Thus, ameliorating the hydrophilicity of the G anodes should be an effect strategy to further improve their performance. Graphene oxide (GO) is single- or few layer graphite oxide which is often used as the precursor of graphene in the chemical synthesis route [10,11]. In GO molecular structure, the contiguous aromatic lattice is interrupted by epoxides, hydroxyls, ketone carbonyls, and carboxylic groups [12]. Within this, the hydroxyls and carboxylic groups endowed GO excellent hydrophilic property and it has been widely used in the hydrophilic modification of filtering membranes [13,14] and fabrics [15]. In this paper, different amounts of GO were doped into the graphene modified layers to elevate the hydrophilicity of the anode surfaces so as to further enhance the performance of the G anodes. The influence of the hydrophilicity variation of the G-GO anodes on the electrogenesis and the organics efficacious utilization in the corresponding MFCs has been investigated in detail.

## 2. Experimental

### 2.1. Chemicals and Materials

Graphene and graphene oxide (GO) was purchased from Suzhou Graphene Nano Tech Co. Ltd. (Suzhou, China). Carbon cloth and polytetrafluoroethylene (PTFE) solution (60 wt %) were purchased from Hesen Electric Co. Ltd., (Shanghai, China). Activated carbon (AC), carbon black (CB), and stainless steel mesh (SSM) were purchased from Xinsen Carbon Co. Ltd. (Shaowu, China), Jinqiushi Chemical Co. Ltd. (Tianjin, China) and Anping Ruishen Mesh Co. Ltd. (Anping, China), respectively. 

### 2.2. Electrode Fabrication

Graphene powder of 10 mg, certain amount of GO powder (1.5 mg, 2.0 mg and 2.5 mg), 1 mL PTFE emulsion (60%) and 1 mL ultrapure water were evenly mixed into paste. Then the pastes were coated on round carbon cloths (3 cm in diameter) and dried at room temperature, respectively. These anodes were named as G, G-GO_0.15_, G-GO_0.2_ and G-GO_0.25_, according to the doped ratios of the GO (GO/G, mg·mg^−1^) in the modified layer (0.15 mg·mg^−1^, 0.2 mg·mg^−1^ and 0.25 mg·mg^−1^). The AC air-cathodes were made by the previously reported rolling-press method [16].

### 2.3. MFC Construction and Operation

Single-chamber cubic-shaped MFC reactors containing a cylindrical anode chamber (28 mL) which was 4 cm long and 3 cm in diameter were used in this work [17]. The anode and the cathode were placed vertically in the two ends of the cylindrical chamber. All the MFC reactors were inoculated with anaerobic sludge (Taihu Newtown Wastewater Treatment Plant, Wuxi, China), operated at 30 °C and fed the medium containing sodium acetate (1 g·L^−1^), phosphate buffer solution (2.77 g·L^−1^ Na_2_HPO_4_·2H_2_O, 11.53 g·L^−1^ NaH_2_PO_4_·12H_2_O, 0.31 g·L^−1^ NH_4_Cl, 0.13 g·L^−1^ KCl), trace mineral (12.5 mL·L^−1^) and vitamin (5 mL·L^−1^) solution [18]. 

### 2.4. Measurement and Analysis

The output voltage (*U*) across the external resistor (1000 Ω) was recorded at 30 min intervals by a data acquisition system (34972A, Agilent Technology Co. Ltd., Santa Clara, CA, USA) connected to a computer. After the electrogenesis of the MFC reached the steady state, a resistance box (ZX79JD, Xian Instruments Company, Xi’an, China) was connected to the MFC and the external resistor was varied from 68 Ω to 33,000 Ω with a time interval of 30 min. The voltages and the electrode potentials at different external resistors were tested by a digital multimeter (UT70B, Uni-Trend Technology Ltd., Dongguan, China). The corresponding current density and output power density were calculated according to the Ohm’s law. The static water contact angles of the anode surfaces were measured by the automatic video optical contact angle measuring system (OCA 40, Dataphysics, Filderstadt, Germany). Chemical oxygen demand (COD) was tested according to the Standard Methods (APHA, 1998) for each cycle and conducted in triplicates for all samples [5]. The coulombic efficiency (*CE*) was calculated by the previously reported method [19]. Electrochemical characterizations were performed in the three-electrode system using the electrochemical workstation (CHI660D, Chenhua Instruments Co. Ltd., Shanghai, China). The anode, Pt wire electrode and the saturated calomel electrode (SCE) were used as working electrode, counter electrode and reference electrode, respectively. The cyclic voltammograms (CVs) were tested at the scan rate of 5 mV·s^−1^ from −0.5 V to 0.5 V in the 50 mM PBS. The electrochemical impedance spectra (EIS) were measured over the frequency range of 0.01~100,000 Hz at open circuit potential and the amplitude was 5 mV [20]. The biomass of anodes was determined by phospholipid analysis according to the previously reported method [21]. The absorbance at 610 nm was then read using a spectrophotometer (UV-1800, Shimadzu, Japan) and the biomass concentration was expressed as the mass of phosphorus per square centimeter of anode (μg·cm^−2^).

## 3. Results and Discussion

### 3.1. Hydrophilicity of G-GO Anodes

**Figure 1:**
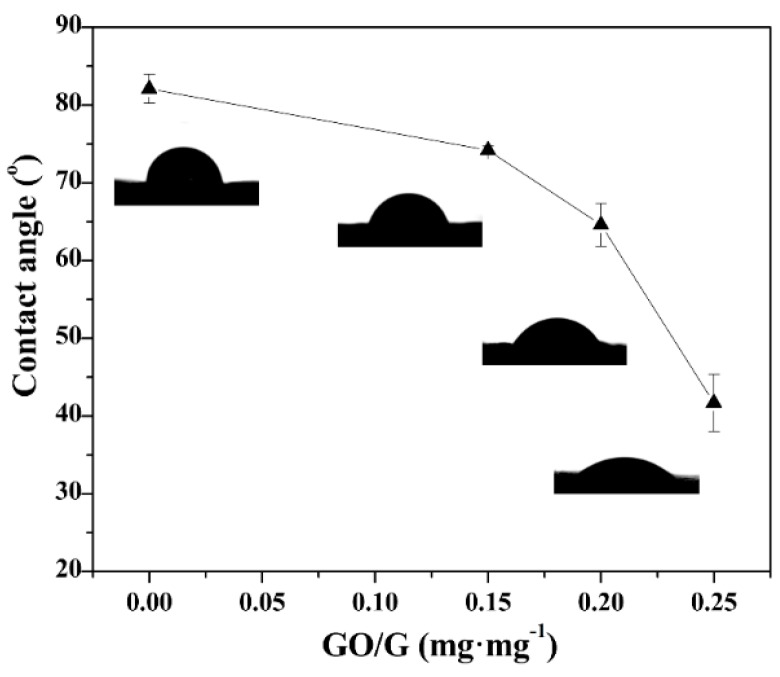
The static water contact angles (*θ*_c_) of the G and G-GO anodes.

The static water contact angles (*θ*_c_) of the G and G-GO anodes were tested to evaluate their hydrophilicity. As illustrated in Figure 1, the *θ*_c_ of the G anode was 82.1 ± 1.88° and with the increase of the GO doped ratio from 0.15 mg·mg^−1^ to 0.2 mg·mg^−1^ and 0.25 mg·mg^−1^, the *θ*_c_ of the G-GO anodes gradually decreased from 74.2 ± 0.52° to 64.6 ± 2.75° and 41.7 ± 3.69° indicating the hydrophilicity improvement of the anode surfaces [22]. 

### 3.2. Performance of G-GO Anodes

**Figure 2:**
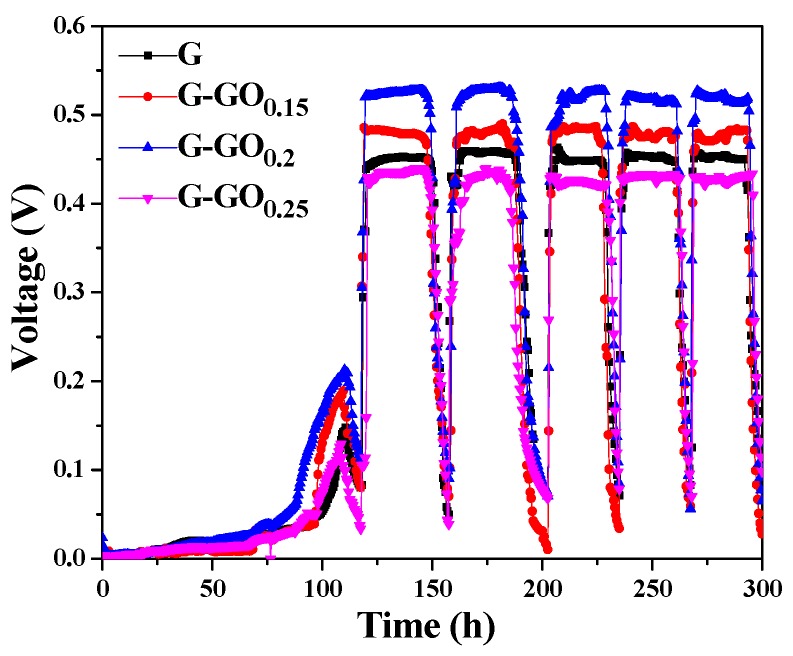
Output voltages of the microbial fuel cells (MFCs) with graphene (G) and G-graphene oxide (GO) anodes.

To investigate the performance of the G-GO anodes, four MFC reactors were run in parallel. As shown in Figure 2, all four MFCs reached steady electrogenesis state after ~119 h, and the average *U* of the MFCs at stable status with the G, G-GO_0.15_, G-GO_0.2_ and G-GO_0.25_ anodes were 0.45 V, 0.47 V, 0.52 V and 0.42 V, respectively. It was worth noting that the MFC with the G-GO_0.2_ anode rather than the G-GO_0.25_ anode exhibited the optimal *U*. This was probably because excessive GO increased the hydrophilicity, but, at the same time, decreased the conductivity or the electrochemical activity of the modified layer.

**Figure 3:**
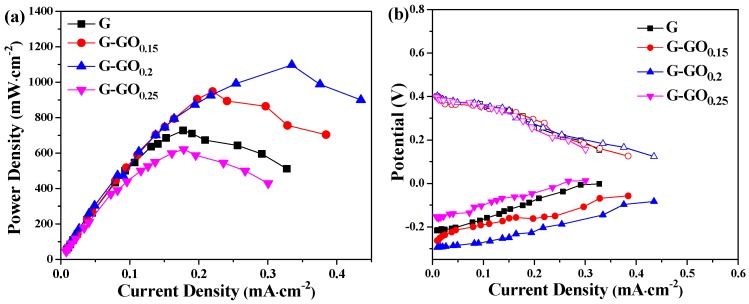
(**a**) Power density curves and (**b**) electrode potentials of the MFCs with G and G-GO anodes.

The output power density and polarization curves were illustrated in Figure 3. As can be seen from Figure 3a, the maximum power density (*P*_max_) of the MFCs with the G anode was 730.72 mW·m^−2^. However, the *P*_max_ of the MFC with the G-GO_0.15_, G-GO_0.2_ and G-GO_0.25_ anodes were 951.08 mW·m^−2^, 1100.18 mW·m^−2^ and 622.18 mW·m^−2^, respectively. The MFC with the G-GO_0.2_ anode exhibited the highest *P*_max_, which was 1.51 times higher than that of the MFC with the G anode. However, the *P*_max_ of the G-GO_0.25_ anode MFC decreased by 14.85% compared with the G anode. Thus it can be seen that a moderate amount of GO doping in the modified layer was the crux for further enhancing the performance of the G anodes. Figure 3b showed the electrode potentials of the different MFCs with SCE as the reference electrodes. The cathode potentials were almost the same while the potentials of anodes were different, indicating that the differences in power densities were mainly caused by the anodes [23].

### 3.3. Substrate Utilization and Coulombic Recovery

**Figure 4:**
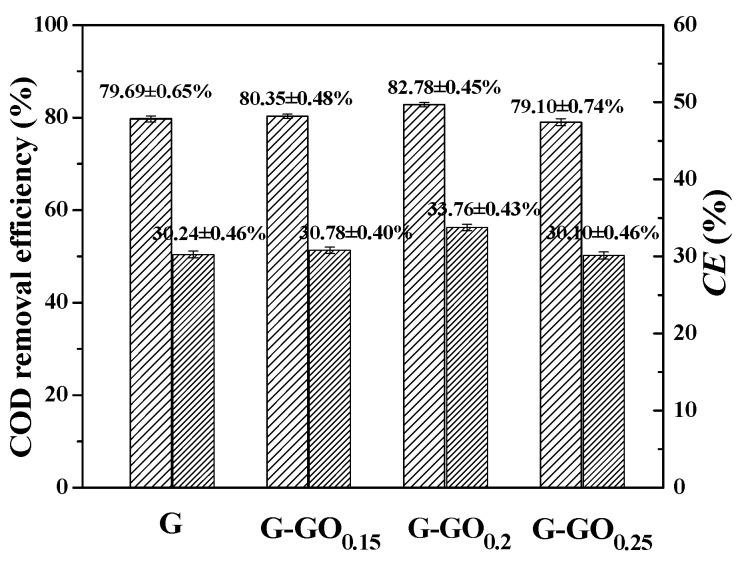
Chemical oxygen demand (COD) removal efficiency and *CE* of MFCs with G and GO anodes.

The COD removal efficiency and the coulomb efficiency (*CE*) of the MFCs with G and G-GO anodes were illustrated in Figure 4. COD removal efficiencies of the G, G-GO_0.15_, G-GO_0.2_ and G-GO_0.25_ MFCs were 79.69 ± 0.65%, 80.35 ± 0.48%, 82.78 ± 0.45% and 79.10 ± 0.74%, respectively. The *CEs* of the corresponding MFCs were 30.24 ± 0.46%, 30.78 ± 0.40%, 33.76 ± 0.43% and 30.10 ± 0.46%, respectively. Similarly, the MFC with the G-GO_0.2_ anode exhibited slightly higher substrates removal and efficacious utilization than other MFCs.

### 3.4. Electrochemical Characterization of G and GO Anodes

**Figure 5:**
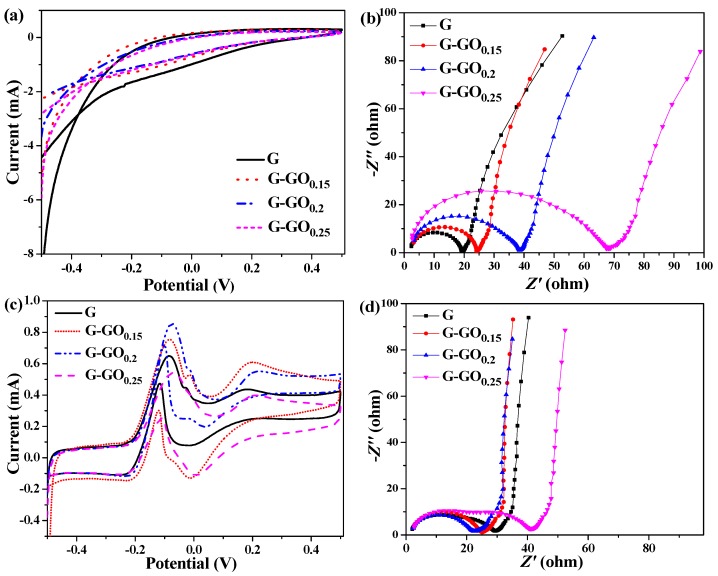
Cyclic voltammograms (CVs) and Nyquist plots of G and GO anodes before (**a**,**b**) and after (**c**,**d**) running.

Cyclic voltammograms (CVs) and electrochemical impedance spectra (EIS) tests were conducted to evaluate the electrochemical activity of the G and G-GO anodes before and after running. As shown in Figure 5a, no redox peak appeared on the CV curves, which indicated that there was no electrochemically active substance on the anodes. With the increase of GO, the current density of the corresponding anodes gradually decreased, suggesting that the doping of GO slightly decreased the double layer capacitance of the anodes [24]. According to Figure 5b, the ohmic resistances (*R*_ohm_) of the anodes were almost the same which was probably because graphene had excellent conductivity and adding small amount of GO has not changed the overall conductivity of the anodes. However, the charge transfer resistance (*R*_ct_) of the anodes gradually increased with the increase of the GO, probably due to the variation of the double layer capacitance. This phenomenon also appeared in the previous reported MnO_2_ modified anodes [25,26]. 

As can be seen from Figure 5c, after running, three couples of redox peaks appeared on the CV curves of the MFCs, which was consistent with the trait of the *G. sulfurreducens* biofilm [27,28]. Within this, the first peak in the forward scan near −0.08 V and the second peak in the reverse scan around −0.22 V might be due to the bio-electrocatalytic redox reaction of acetate [29]. The oxidation peak currents of the G-GO_0.15_ and the G-GO_0.2_ anode were obviously higher than that of the G and the G-GO_0.25_ anode, indicating their relatively higher anodic biocatalysis. The other couple of peaks were probably due to the existence of electrocatalytic active substances on the outer membranes of the exoelectrogens (like the cytochrome, *OmcB*, *OmcE* or *OmcS*) [30]. Figure 5d showed the Nyquist plots of different anodes after running. Compared with the Nyquist plots in Figure 5b, the change of the *R*_ohm_ for the anodes was negligible while the *R*_ct_ of the anodes obviously decreased after running, which revealed the forming of electrochemical active biofilms. The *R*_ct_ of the G-GO_0.15_ and G-GO_0.2_ were 23.07 Ω and 20.56 Ω, respectively, which were much smaller than that of the G anode (28.14 Ω) indicating that the extracellular electron transfer (EET) in the biofilms of G-GO anodes were greatly enhanced [31,32]. However, the *R*_ct_ of the G-GO_0.25_ anode increased to 39.02 Ω, suggesting that excess GO in the modification layer adversely affected the EET.

### 3.5. Anode Biomass

In MFC, substrates are oxidized by the electrochemically active microorganisms growing on the surface of the anode to generate electrons, and then these electrons transfer into the external circuit and finally arrive at the cathode to be consumed by electron accepters to complete the electricity generation circulation [33]. Therefore, the more electrochemically active microorganisms attached on the anode surface, the more electrons generated and the higher *P*_max_ of the MFC. As shown in Table 1, the amount of microbial biomasses on the anode surfaces of the MFCs were characterized by the phospholipid concentrations [21]. The phospholipid concentration of the biofilm on the G anode was 6.68 ± 0.57 μg∙cm^−2^. The G-GO_0.15_ and G-GO_0.2_ anodes showed obvious higher phospholipid concentrations. As the GO doped ratio increased from 0.15 mg·mg^−1^ to 0.2 mg·mg^−1^, the phospholipid concentration on the corresponding anodes ascended from 6.86 ± 0.56 μg∙cm^−2^ to 7.07 ± 0.56 μg∙cm^−2^, respectively. Hence, it can be seen that the doped GO enhanced the hydrophilicity of the anodes and evidently promoted the microbial attachment and growth on the anodes. However, excess GO doped into the modified layer obviously decreased the double layer capacitance of the anode, hindered the EET between the biofilm and the anode, and finally limited the reproduction of the bacteria on the anode surface. Therefore, the phospholipid concentration of the biofilm on the G-GO_0.25_ anode was lower than that of the G anode. 

## 4. Conclusions

The hydrophilicity of the G anodes was elevated by doping small amounts of GO. As the GO doped ratio increased from 0.15 mg·mg^−1^ to 0.2 mg·mg^−1^ and 0.25 mg·mg^−1^, the static water contact angle (*θ*_c_) of the G-GO anodes decreased from 74.2 ± 0.52° to 64.6 ± 2.75° and 41.7 ± 3.69°, respectively. Under the combined influence of the hydrophilicity and the double layer capacitance, the G-GO_0.2_ anode with medium graphene doping ratio achieved the greatest performance. The *P*_max_ of the MFC with G-GO_0.2_ anode was 1100.18 mW·m^−2^ which was 1.51 times higher than that of the MFC with the G anode. In conclusion, doping a moderate amount of GO was feasible to further improve the performance of the G anodes and boost the output power of MFCs.

## Figures and Tables

**Table 1:** The phospholipid concentrations of G and G-GO anodes.

Anodes	G	G-GO_0.15_	G-GO_0.2_	G-GO_0.25_
Phospholipid concentration (μg·cm^−2^)	6.68 ± 0.57	6.86 ± 0.56	7.07 ± 0.56	6.11 ± 0.57
